# CRISPR-Cas9-mediated homology-directed repair rescues the induced bone marrow failure in *Fancc*^−/−^ mice

**DOI:** 10.1016/j.omtn.2026.102912

**Published:** 2026-03-23

**Authors:** Hemavathy Harikrishnan, Mahesh Lamsal, Ka-Kui Chan, Junping Zhang, Jiahe Tian, Kwadwo Fosu, Hong Phuong Nguyen, D. Wade Clapp, Elizabeth A. Sierra Potchanant, Reuben Kapur, Weidong Xiao, Ngoc Tung Tran

**Affiliations:** 1 Herman B Wells Center for pediatric research, Department of Pediatrics, Indiana University School of Medicine, Indianapolis, IN, USA; 2 Department of Computer Science, Cornell University, Ithaca, NY, USA

**Keywords:** MT: RNA/DNA editing, CRISPR-Cas9, homology-directed repair, bone marrow failure, fanconi anemia, FANCC, gene therapy, gene correction

## Abstract

Fanconi anemia (FA) is a rare recessive genetic disorder resulting from mutations in genes in the FA-DNA repair pathway. Among its subtypes, FA complementation group C (*FANCC*) is associated with particularly severe hematologic and developmental manifestations. Gene therapy targeting autologous hematopoietic stem/progenitor cells (HSPCs) from FA patients represents a promising curative strategy for FA-associated bone marrow failure (BMF), potentially circumventing the limitations of allogeneic transplantation. Despite substantial progress in developing gene therapy for FA group A (*FANCA* mutations), therapeutic strategies for FA group C have received comparatively little attention. Although FA-deficient cells are believed to exhibit compromised homologous recombination (HDR), our reporter assay demonstrated that *Fancc*^−/−^ mouse HSPCs retain HDR activity, supporting the feasibility of precise gene editing. Building on this finding, we established a CRISPR-Cas9/AAV6-HDR platform to integrate Fancc cDNA into its endogenous locus while minimizing off-target effects and AAV integration. Correction of *Fancc*^−/−^ HSPCs restored *Fancc* expression, rescued colony-forming capacity, and improved cellular viability. Importantly, transplantation of corrected HSPCs into *Fancc*^−/−^ mice conferred resistance to mitomycin C-induced BMF, demonstrating durable *in vivo* functional correction. Collectively, these results establish CRISPR-Cas9-mediated HDR as a viable and potential therapeutic strategy for FA group C.

## Graphical abstract

**Figure:**
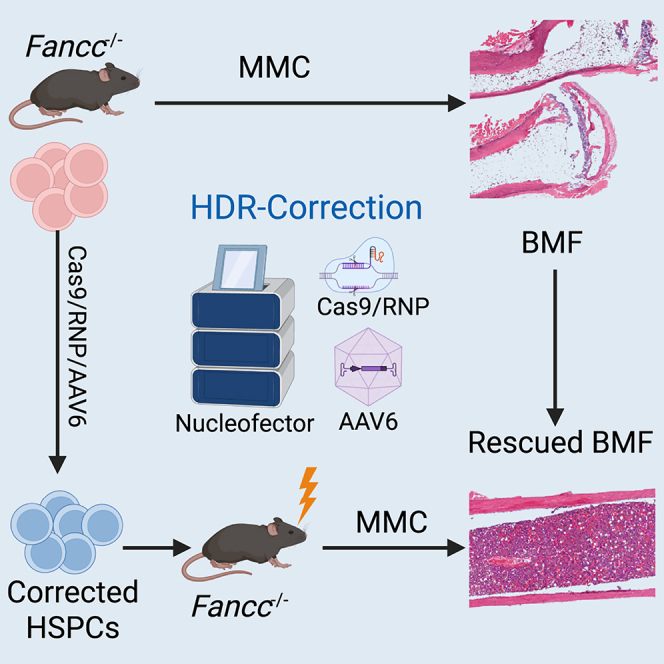

## Teaser

Harikrishnan and colleagues exploited the Cas9RNP/AAV6 to correct the *Fancc* gene in hematopoietic stem progenitor cells isolated from *Fancc*^−/−^ mice with minimal off-target activities and AAV integration. Of note, corrected stem cells restored the expression of *Fancc* gene and protected *Fancc*^−/−^ mice from mitomycin C-induced bone marrow failure.

## Introduction

Fanconi anemia (FA) is a severe recessive monogenic disorder caused by mutations in the FA pathway, with approximately 90% of disease-associated mutations occurring in three core complex genes: FA complementation group C (*FANCC*), *FANCA*, and *FANCG*.^1^ FA patients face congenital abnormalities, cancer predisposition, and progressive bone marrow failure (BMF), which is the leading cause of early mortality in pediatric patients. FA group C patients exhibit typical, but more severe symptoms than other subtypes. Lentivirus-based gene therapy to deliver functional *FANCA* gene in autologous FA-HSPCs showed promising outcomes in rescuing FA-mediated BMF and overcoming the limitations of allogeneic bone marrow transplantation.^2^ Of note, corrected FA-HSPCs engrafted into patient without conditioning, demonstrating the feasibility of precise gene correction in autologous HSPCs to rescue the BMF.^3^ Lentivirus-based gene therapy for FA group C patients harboring mutations in *FANCC* gene is currently under development.

Here, we established the CRISPR-Cas9-mediated homology directed repair (HDR) system to restore the functions of mouse *Fancc*^−/−^ HPSCs. Using the HDR system, we were able to integrate the functional *Fancc* gene efficiently into an endogenous promoter and restore the expression of FANCC in mouse *Fancc*^−/−^ HSPCs. Of note, corrected HSPCs after transplantation prevented BMF in *Fancc*^−/−^ mice upon the exposure to mitomycin C (MMC). In summary, we demonstrated the feasibility of CRISPR-Cas9-mediated HDR to prevent MMC-induced BMF in *Fancc*^−/−^ mice, providing a strong preclinical gene therapy model for FA group C patients.

## Results

### *Fancc*^−/−^ mouse is a faithful model for CRISPR-Cas9-mediated gene therapy

The *Fancc*^−/−^ mouse model generated by replacing exon 8 with a neomycin cassette, resulting in complete loss of Fancc function, has been previously described.^4^^,^^5^^,^^6^^,^^7^ Unlike human FA patients, *Fancc*^−/−^ mice do not spontaneously develop severe aplastic anemia.^8^ Nevertheless, intrinsic defects of *Fancc*^−/−^ hematopoietic stem and progenitor cells (HSPCs) have been demonstrated, particularly under conditions of replicative stress.^9^ In competitive transplantation assays, *Fancc*^−/−^ HSPCs exhibit markedly reduced short- and long-term repopulating ability.^4^^,^^5^^,^^6^ Importantly, these functional impairments could be rescued by reintroducing a functional *Fancc* gene via retroviral transduction,^10^ confirming that *Fancc* is essential for maintaining HSPC function *in vivo*.

**Figure 1:**
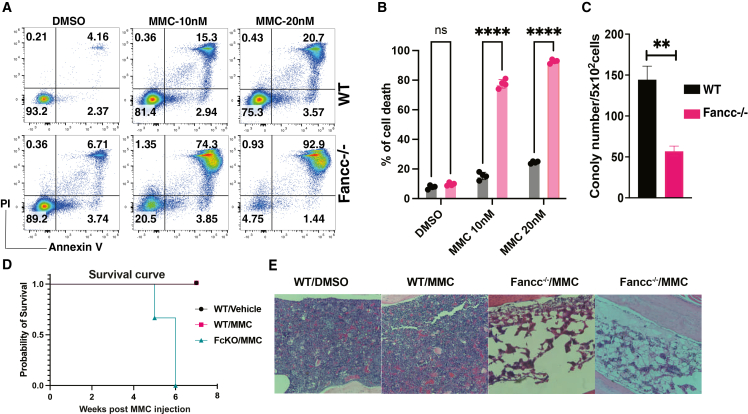
Mitomycin C hypersensitivity of *Fancc*^−/−^ mice (A) Flow cytometry presents the apoptosis assay of respective HSPCs treated with different concentrations of MMC. Cells were stained with Annexin V and propidium iodide (PI). (B) Graph summarizes data in (A), and data were presented as a mean of percentage of cell death from three independent experiments. (C) Bar graph shows the number of forming colonies per seeded 500 HSPCs isolated from indicated mice. Data were presented as a mean of colony numbers from 3 independent experiments. (D) Kaplan-Meier curves depict the survival of indicated mice upon treated with vehicle control or MMC (0.3 mg/kg). (E) H&E staining of bone sectioning isolated from treated mice in (D). One-way ANOVA test and unpaired *t* test were used to compare the different between experimental groups in (B) and (C), respectively. ∗∗*p* < 0.01, ∗∗∗∗*p* < 0.0001, ns, no significance.

To further validate these defects, we isolated Sca1^+^ cells from *Fancc*^−/−^ mice and wild-type (WT) littermates and exposed them to increasing doses of MMC, which induces DNA interstrand crosslinks (ICLs) that FA-deficient cells are unable to repair. As expected, *Fancc*^−/−^ Sca1^+^ cells were hypersensitive to MMC compared to WT cells (Figures 1A and 1B) and displayed significantly reduced colony-forming capacity (Figure 1C). To model BMF *in vivo*, *Fancc*^−/−^ and WT mice were treated with five doses of MMC (0.3 mg/kg). While WT mice remained resistant, all *Fancc*^−/−^ mice succumbed to treatment (Figure 1D). Histological analysis of bone sections revealed preserved marrow cellularity in WT and MMC-treated WT mice, whereas MMC-treated *Fancc*^−/−^ mice exhibited a hypocellular marrow phenotype indicating BMF (Figure 1E). These results are consistent with prior reports^11^ demonstrating that *Fancc*^−/−^ mice are highly sensitive to MMC and prone to BMF.

### CRISPR-Cas9-mediated HDR to restore *Fancc* gene in *Fancc*^−/−^ HSPCs

We previously established a culture system that enriches mouse Lin^−^Sca1^+^cKit^+^ (LSK) cells from Sca1^+^ progenitors, optimized for efficient CRISPR-Cas9-mediated gene editing via HDR. Using this platform, we successfully corrected the *Rag2* gene in *Rag2*^−/−^ mice and restored B/T cell development *in vivo*.^12^ Here, we applied this system to correct the *Fancc* gene in *Fancc*^−/−^ HSPCs. Given that FA-deficient cells are generally impaired in HDR, we first assessed HDR activity in *Fancc*^−/−^ HSPCs using a reporter in which a T2A-mCherry cassette was integrated into the last exon of Lmnb1.^12^ Successful HDR resulted in mCherry expression, allowing quantification of repair efficiency. Compared with WT cells, *Fancc*^−/−^ HSPCs exhibited reduced HDR activity; however, inhibition of the non-homologous end joining (NHEJ) pathway by exposure to the DNA-PKC inhibitor, M3184, significantly enhanced HDR in these cells (Figure S1). These findings demonstrate that HDR remains functional, though less efficient, in *Fancc*^−/−^ HSPCs, enabling us to pursue targeted correction of the *Fancc* locus.

**Figure 2:**
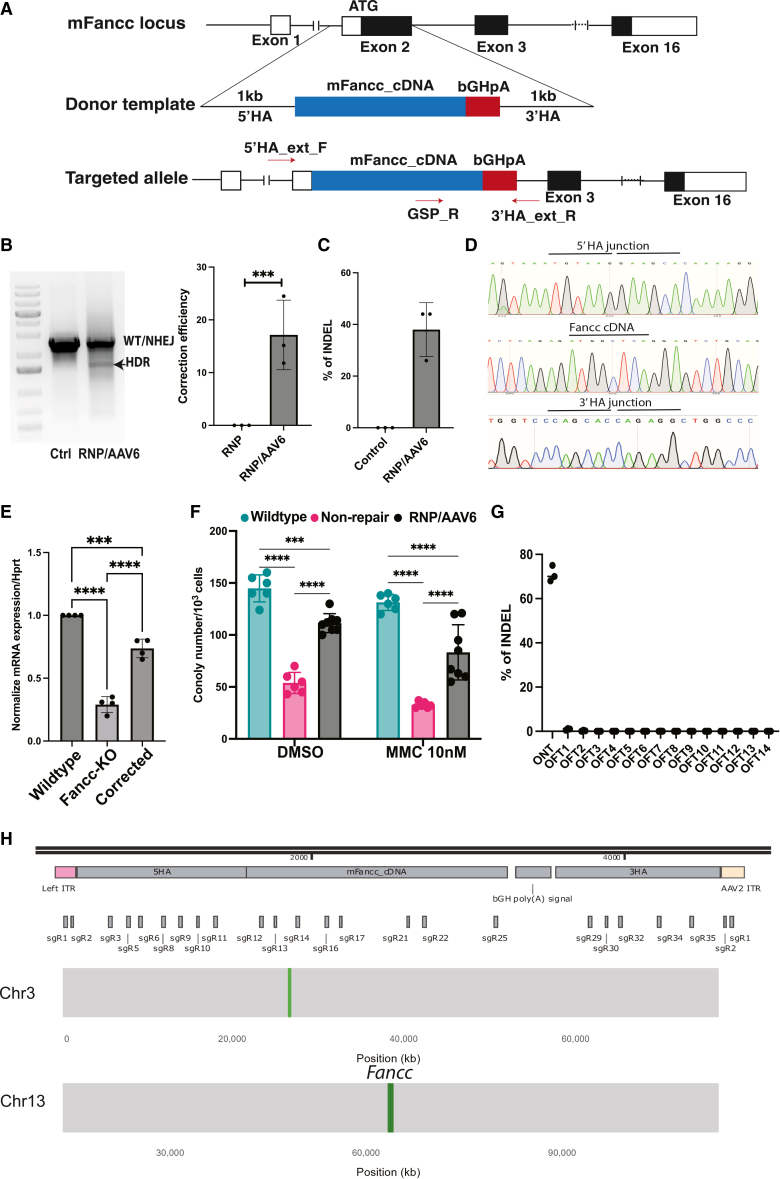
Gene correction of *Fancc* gene in *Fancc*^−/−^ HSPCs (A) Targeting strategy to restore mouse *Fancc* gene. (B) Gel picture showing the results of 3-primer PCR system that can detect WT/NHEJ and HDR events. Graph (right) summarizes the means of correction efficiency from 3 independent experiments. (C) Graph summarizes the percentage of indel detected in at the targeted sites in the corrected stem cells. (D) Sanger sequencing to confirm the precise integration of *Fancc* cDNA into the endogenous locus. (E) Bar graph depicts the expression of *Fancc* at the transcriptional level determined by real-time PCR. *Hprt* was used as a house keeping gene. Data were presented as a mean of normalized mRNA expression from four independent experiments. (F) Graph summarizes the number of forming colonies from indicated cells treated with either DMSO or 10 nM MMC. Data were presented as a mean of colony number from 6 independent experiments. (G) Graph presents the percentage of indel detected by sequencing in the 14 potential off-target site (OFT) and the on-target site (OT). (H) AAV integration assay. Upper scheme shows the positions of designed sgRNAs targeting different portion of AAV genome. AAV integration sites on indicated chromosomes were presented as green vertical lines. One-way ANOVA test was used to compare the differences between experimental groups in (E and F). Unpaired *t* test was used to compare in the B and C. ∗∗∗*p* < 0.001, ∗∗∗∗*p* < 0.0001.

Our strategy was to integrate codon-optimized *Fancc* cDNA into the endogenous locus (Figure 2A). Several small guide RNAs (sgRNAs) targeting exon 2 of *Fancc* were designed and validated (Figure S2A). All sgRNAs effectively targeted exon 2, with sg6 yielding the highest HDR activity and highest restoration of *Fancc* expression compared with sg2 (Figure S2B and C). Consequently, sg6 was selected for subsequent experiments. The donor template consisted of *Fancc* cDNA followed by a polyA signal, flanked by 1-kb homology arms (HAs), and was cloned into an adeno associated virus serotype 6 (AAV6) vector for delivery. CRISPR-Cas9 ribonucleoprotein (RNP) complexes were introduced into *Fancc*^−/−^ HSPCs by electroporation, followed immediately by AAV6-mediated donor delivery. Three days after editing, HDR efficiency was evaluated.

To quantify HDR, we developed a three-primer PCR assay capable of distinguishing unedited/NHEJ alleles from HDR-edited alleles (Figure 2A). Quantification of the PCR band intensity on gels indicated that we achieved in average of approximately 20% HDR efficiency (Figure 2B). We also isolate the upper band and sent out for sequencing to evaluate the indel at the targeted site. As a result, we observed approximately 40% frequency of indel (Figure 2C). To assess the integrity of the insertion at the junction of 5′HA and 3′HA, we used one primer annealed to outside of 5′HA or 3′HA and one primer annealed to cDNA. With this design, we only amplified targeted allele but not the episomal AAV. PCR products were sent out for sequencing verified precise integration of *Fancc* cDNA into the endogenous locus (Figure 2D). Notably, HDR-corrected cells restored *Fancc* mRNA expression to near WT levels (Figure 2E), demonstrating successful correction of the genetic defect.

To evaluate potential off-target activities of our gene-editing system, we used CrispRGold^13^ to computationally predict sites with sequence mismatches to sg6. Of the 14 predicted sites, 13 were classified as high-risk candidates, all located within intronic or intergenic regions (Table S1). These regions were PCR-amplified and subjected to amplicon sequencing. Analysis revealed only background levels of indels (<0.1%) across all predicted sites (Figure 2G).

We next assessed AAV integration in corrected stem cells. Edited cells were cultured for an additional 21 days to eliminate residual episomal AAV. To detect integrated AAV sequences, we designed an array of sgRNAs targeting distinct regions of the AAV genome, including the inverted terminal repeat (ITR), 5′HA, *Fancc* cDNA, and 3′HA (Figure 2H). Genomic DNA was isolated from edited cells, treated with Cas9/sgRNA complexes *in vitro*, and processed for nanopore long-read sequencing. Sequencing reads were aligned against both the AAV reference and the mouse genome to identify integration sites. As expected, we detected targeted AAV integration at the *Fancc* locus on chromosome 13. In addition, we observed an integration event on chromosome 3 (Figure 2H). Notably, these integration sites did not correspond to the predicted off-target sites (Table S1), suggesting random integration of AAV.

### HDR-corrected *Fancc*^−/−^ HSPCs prevent MMC-induced BMF

We next evaluated the functional capacity of corrected HSPCs *in vitro* using colony-forming unit (CFU) assays in combination with MMC treatment. Wild-type (WT), non-corrected, and corrected HSPCs were exposed to DMSO or 10 nM MMC for 2 h and subsequently plated for CFU assays. As expected, non-corrected *Fancc*^−/−^ HSPCs displayed markedly impaired colony-forming ability, whereas corrected HSPCs exhibited partial recovery, approaching the capacity of WT cells. Importantly, corrected HSPCs showed greater resistance to MMC compared to non-corrected controls (Figure 2F).

We next investigated the *in vivo* functionality of corrected HSPCs. We confirmed that *Fancc*^−/−^ mice were hypersensitive to MMC and developed BMF upon repeated doses of MMC (Figure 1). We therefore used this stringent assay to assess the *in vivo* function of corrected HSPCs.

**Figure 3:**
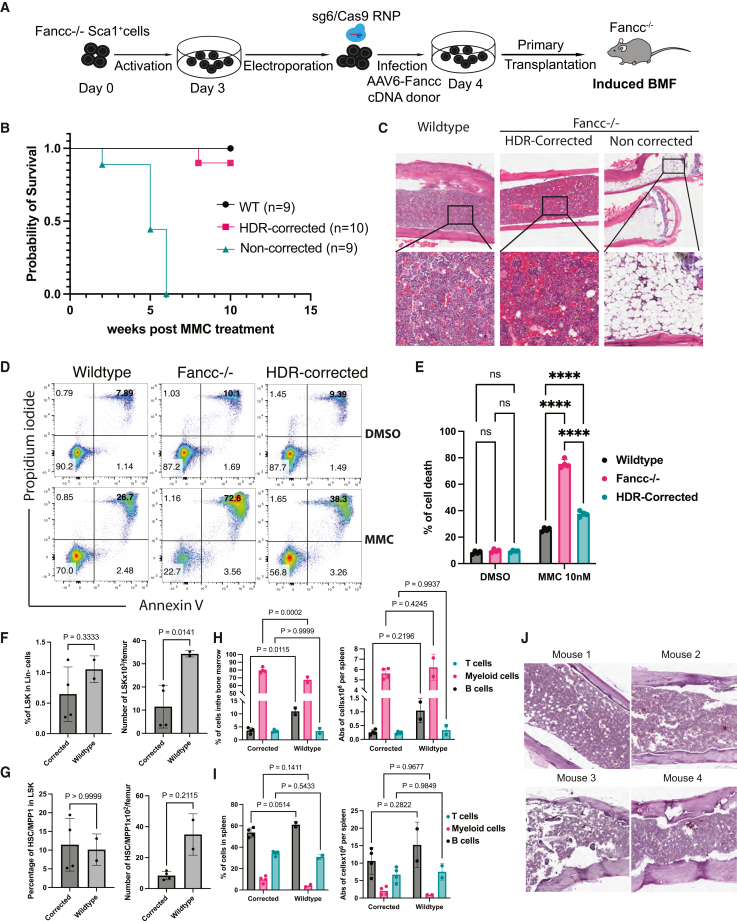
Corrected HSPCs protect *Fancc*^−/−^ from MMC-induced BMF (A) Experimental scheme. (B) Kaplan-Meier curves present the survival of indicated mice upon treatment with 5 dose of MMC (0.3 mg/kg), *n* numbers were indicated. (C) H&E staining of bone sections isolated from treated mice in (B). (D) Flow cytometry data depicts the apoptosis of cells isolated from indicated mice upon treated with 10 nM MMC. (E) Bar graph summarizes data from (D). Graphs show the percentage and absolute number of LSK (F) and HSC/MPP1. (G) Cell compartments in the bone marrow were isolated from the 2^nd^ transplanted mice. Bar graphs depict the percentage and absolute number of B, T, and myeloid cells in the bone marrow (H) and spleen (I) isolated from the 2^nd^ transplanted mice. Two-way ANOVA test was used to compare the differences between corrected and WT mice. *p* values were showed on the graph. (J) H&E staining of bone sections isolated from secondary transplanted mice following MMC treatment. One-way ANOVA test was used to compare the differences between experimental groups in (E). ∗∗∗∗*p* < 0.0001; ns, not significant. *t* test was used to compare the differences between groups in F-I, *p* values were displayed.

As M3184 might lead to genome instability,^14^ we performed the gene correction without using this inhibitor. One day post-gene correction, non-corrected or corrected HSPCs were transplanted into lethally irradiated *Fancc*^−/−^ mice. All recipients survived transplantation, demonstrating successful engraftment and reconstitution. Three months post-transplantation, WT mice and *Fancc*^−/−^ recipients of non-corrected or corrected HSPCs were challenged with MMC (Figure 3A). As expected, all WT mice survived (*n* = 9), whereas all *Fancc*^−/−^ mice receiving non-corrected HSPCs (*n* = 9) succumbed after five MMC doses. Remarkably, 90% of *Fancc*^−/−^ mice transplanted with corrected HSPCs (9 out of 10) survived (Figure 3B). Bone marrow histology confirmed normal cellularity in WT and corrected HSPC recipients, whereas non-corrected HSPC recipients showed severe hypocellularity (Figure 3C). Furthermore, bone marrow cells isolated from surviving corrected HSPC recipients displayed resistance to MMC nearly equivalent to WT (Figures 3D and 3E).

To further assess the long-term functional capacity of corrected HSPCs, bone marrow cells from four surviving primary recipients (4 corrected mice and 2 WT mice) were transplanted into secondary *Fancc*^−/−^ hosts. Three months after transplantation, secondary recipients were challenged with five doses of MMC. Notably, all secondary transplanted mice survived MMC treatment and did not develop BMF. Following MMC challenge, we analyzed the cellular composition of the bone marrow and spleen in these mice. In the bone marrow, we observed a significant reduction of absolute cell numbers of LSK and HSC/MPP1 (CD48^−^CD150^+^LSK) compartments in corrected mice compared with WT controls (Figures 3F, 3G, and S3A). We next examined mature hematopoietic populations. In both bone marrow (Figures 3H and S3B) and spleen (Figures 3I and S3C), corrected mice exhibited a significantly lower proportion of B cells and a corresponding increase in myeloid cells relative to WT mice; however, the absolute numbers of B cells and myeloid cells were not significantly different between the groups. There is a trend of myeloid bias in corrected mice compared to WT counterpart. No differences were observed in T cell populations between corrected and WT mice. Histological analysis of bone marrow sections from secondary recipients demonstrated normal marrow cellularity, further confirming preserved hematopoietic function following secondary transplantation (Figure 3J). Collectively, these results demonstrate that HDR-corrected HSPCs retain robust long-term repopulating ability and confer protection against stress-induced BMF *in vivo*.

## Discussion

In this study, we demonstrate that CRISPR-Cas9-mediated HDR can effectively correct the *Fancc* gene in HSPCs derived from *Fancc*^−/−^ mice. We achieved efficient correction and, notably, partial restoration of *Fancc* expression was observed in the edited HSPCs. Importantly, these corrected cells regained functional competence, as evidenced by both *in vitro* assays and *in vivo* transplantation. Furthermore, the corrected HSPCs conferred resistance to MMC-induced BMF, a hallmark phenotype of FA. Our results reveal that gene correction of *Fancc* in HSPCs is not only feasible but also functionally meaningful. Even partial correction was sufficient to restore critical aspects of hematopoietic function in the FA model. This aligns with the notion that a relatively small pool of corrected stem cells can provide significant hematologic benefit in FA patients, as previously observed in cases of spontaneous genetic reversion.^15^

Various gene editing tools have been implemented for FA model, almost exclusively focusing on FA group A. While non-homologous end joining (NHEJ)-mediated INDELs have been shown to partially restore the reading frame of mutated FA genes and rescue the proliferative defects of patient-derived stem cells,^16^ the *in vivo* function of *FANCC*-edited stem cells has yet to be demonstrated. Thus, precise correction of *FANCC* mutations is essential for fully restoring the functions of FA-HSPCs. Base editors^17^^,^^18^ and prime editors^19^ have been developed as double-stranded break (DSB)-independent approaches to efficiently correct point mutations and introduce small insertions, respectively. Base editors efficiently corrected common mutations in *FANCA* gene.^20^^,^^21^ However, these systems have limitations when repairing multiplex mutations or replacing/inserting long DNA sequences, which necessitate a personalized approach for each patient. Recently, PASTE,^22^ a combination of prime editor and integrase, and twin primer editors (PASSIGE, evoPASSIGE, and eePASSIGE)^23^ was developed to insert a large sequence into the genome without generating DSBs. However, these platforms are in their early development stages and demonstrate low efficiency in primary cells.

While promising, our system is associated with certain limitations. First, the introduction of DSBs via CRISPR-Cas9 can lead to considerable cytotoxicity and genomic mutations, particularly in human FA-HSPCs, which are known to have impaired DNA repair capacity and sustained high DNA damage.^15^ AAV6 is known to have a cell-cycle arrest effect and reduced the engraftment of edited cells when used as a donor template source.^24^ High dose of AAV6 also can lead to cell death. Optimizing the virus-free donor template might bring a benefit to improve the functions of corrected FA-HSPCs. Of note, using lipid nanoparticle instead of electroporation to deliver the CRISPR-Cas9 system improves the viability and functions of edited HSPCs.^25^ Due to the fragile of FA-HSPCs, an optimal system that can mediate efficient HDR and minimal toxicity to edited cells is an urgent need. It would require a combination of many factors: delivery method, donor template source, and other known factors that can improve survival and functions of FA-HSPCs. While mouse HSPCs tolerated editing reasonably well in our study, extrapolation to the human system requires caution.

Second, we used germline *Fancc*^−/−^ mice as a host for corrected HSCPs, thus MMC treatment has a broad effect on these mice, not limited to the hematopoietic system. Although, corrected HSPCs prevented the BMF and kept mice alive post-MMC exposure, we observed remarkable decline in health of treated mice. Future studies should utilize either mice with hematopoietic-specific *Fancc* loss or a stress system specific to hematopoiesis. Further, future work should aim to optimize gene editing platforms to minimize DSB-induced toxicity. Emerging technologies such as spacer-nick,^26^ base editing, or prime editing, which do not require DSBs, may offer safer alternatives for FA gene therapy. Additionally, expanding this strategy to human *FANCC*^−/−^ HSPCs will be a critical next step. Lastly, long-term follow-up studies are needed to assess the durability of corrected HSPC engraftment and function, as well as the potential for off-target effects or clonal dominance.

## Materials and methods

### Targeting vector construction and sgRNAs

All sgRNAs were purchased from Synthego, sequences of these sgRNAs and other oligos were listed in the Table S2. To generate donor template, 1-kb 5′ HA, a promoterless and codon-optimized *Fancc* cDNA followed by bovine polyA signal, and 1-kb 3′HA were synthesized by GeneArt (Invitrogen). Silent mutations were added at and around the protospacer site to prevent Cas9-mediated cleavage. The DNA fragment was cloned into AAV vector. AAV6 carrying donor template were produced and tittered as described before.^27^

### Gene editing in mouse HSPCs

Gene editing of mouse HSPCs were performed as described before^12^ with some modifications. Briefly, Sca1^+^ cells were isolated from bone marrow of *Fancc*^−/−^ mice using Sca1 enrichment kit (Miltenyi Biotec). Cells were cultured for 2 days in StemSpan SFEM II medium (STEMCELL Technologies) supplied with mouse SCF (50 ng/mL), mouse TPO (50 ng/mL), mouse Flt3L (50 ng/mL) and human IL-11 (50 ng/mL) (PeproTech). Cultured cells were harvested, washed once with PBS, and subjected to gene correction. RNP complex containing Cas9 protein (50 pmol, IDT) and synthesized sgRNA (100 pmol) were electroporated into 2 × 10^5^ cells using EO-100 electroporation program (Amaxa 4D, Lonza). Electroporated cells were immediately transduced with AAV6 carrying donor template (10^6^ genome copy per cell). Corrected cells will be collected at different time points for different functional assays.

### Quantification of editing events

To quantify gene-editing outcomes, we developed a three-primer PCR strategy consisting of two primers annealing to endogenous genomic sequences outside of the HAs (5HA and 3HA) and a third primer annealing within the donor cDNA. With this system, episomal AAV will not be amplified. Using this assay, non-edited samples yield a single PCR product (∼2.5 kb). In contrast, Cas9 RNP/AAV6-treated samples generate two PCR products: a ∼2.5 kb band corresponding to non-edited or indel-containing alleles and a ∼1.6 kb band representing precise HDR-mediated integration. Both PCR products were excised and subjected to sequencing to quantify indel formation at the target site and to assess the fidelity of donor integration at the 5′ and 3′ HA junctions. HDR efficiency was calculated based on the relative band intensities quantified from gel electrophoresis.

### AAV integration assay

Genomic DNA was fragmented via (cat. no. 520079, Covaris) at 6,000 rpm 60 s to get approximate 10-kb DNA fragments. Fragmented DNA were cleavage by Cas9/sgRNAs using the GenCrispr sgRNA Screening Kit (cat. no. L00689), following manufacturer’s protocol. Samples were purified by AMPure XP beads for native adaptor ligation following the NEB Blunt/TA Ligase Master Mix. Finally, the ligation product was purified by AMPure XP beads for subsequent nanopore sequencing.

Sequencing data was processed using Oxford Nanopore EPI2ME Labs Nextflow workflows. Reads were aligned via wf-alignment (v.1.2.1) using Minimap2 (v.2.26-r1175) with override_basecaller_cfg set to dna_r10.4.1_e8.2_400bps_hac@v4.3.0. Variants were called through wf-human-variation (v.2.4.1), employing Sniffles2 (v.2.07-epi2me) for structural variants (SVs), Clair3 (v.1.0.8) for single nucleotide polymorphisms (SNPs), and CNV kit (v.0.9.10) with 5 kb windows for copy number variations (CNVs).

A combined reference genome was constructed by concatenating the mouse reference genome (GRCm39) with the pAAV_mFancc_cDNA-sgRNA vector sequence and indexed using BWA-MEM (v.0.7.17)^28^ and SAMtools (v.1.13).^29^ Nanopore-sequenced reads in BAM format were converted to FASTQ format using SAMtools and re-aligned to the combined reference genome using BWA-MEM. The resulting alignments were converted to sorted BAM format and indexed using SAMtools. Alignment quality for each sample was assessed by mapping rates and read counts using SAMtools flagstat.

To identify AAV integration sites, AAV-host junction reads were identified in R (v.4.4.1) using the GenomicAlignments (v.1.40.0)^30^ and Rsamtools (v.2.20.0) packages. Reads aligned to the AAV reference sequence were extracted from each sample’s BAM file. For each AAV-aligned read, alignments to host chromosomes were then identified by parsing supplementary alignment (SA) tags. The selected junction reads indicated potential AAV integration sites, and their AAV alignment co-ordinates and host chromosome and genomic co-ordinates were extracted using CIGAR string parsing.

### *In vitro* functional assays

Sca1^+^ cells (corrected, non-corrected, and WT) will be treated with MMC (10 nM) or DMSO control for 2 h. Treated cells will be seeded in the MethoCult M3434 (STEMCELL Technologies) medium for colony-forming assay following the manufacture’s protocol. Number of colonies was counted at day 10. Treated cells were stained with Annexin V-FITC (BioLegend) and analyzed the apoptosis using flow cytometry (Attune NxT, Thermo Fisher Scientific).

### Induced BMF

*Fancc*^−/−^ mice were bred and maintained in house at Indiana University. All the experiments were performed according to the guidelines of the Institutional Animal Care and Use Committee.

Corrected/non-corrected Sca1^+^ cells were transplanted into lethally irradiated *Fancc*^−/−^ mice (2–3 months old). To induce BMF, 3 months post-transplantation, mice were treated with 5 doses of MMC (0.3 mg/kg), once per week. WT mice were used as positive controls that did not develop BMF. To check the cellular structure in the bone marrow, tibias, and femurs were isolated from treated mice. Bones were fixed, decalcified, paraffin-embedded, sectioned, and stained with H&E. Stained sections were captured by microscope.

To check the hematopoietic cells in 2^nd^ transplanted mice, we isolated cells from bone marrow and spleen and stained with antibodies. Cells were analyzed by the Attune NxT Flow Cytometer (Thermo Fisher Scientific). All antibodies were purchased from BioLegend (Table S3).

## Data and code availability

All data are included in the paper or supplemental information. Additional information will be provided upon requested to the corresponding author.

## Acknowledgments

We thank the Preclinical Modeling and Therapeutics Core (PMTC) for the help of carrying out mouse works. We acknowledge the Flow Cytometry Core facility for assisting with flow cytometry-related works. This work is supported by funds from 10.13039/100016606Riley Children Foundation, 10.13039/100000002NIH/R01DK139505 (to N.T.T. and R.K.) and 10.13039/100000002NIH/R21HL168669 and 10.13039/100000005Department of Defense (BM220005) to N.T.T.

## Author contributions

N.T.T. conceived and developed the project; N.T.T., H.H., M.L., K.-K.C., H.P.N., and K.F. designed, performed, analyzed experiments, and interpreted data; W.C., E.A.S.P., and R.K. assisted with *Fancc* mouse experiments; J.P.Z, J.T., and W.X. contributed into AAV integration assay; N.T.T. wrote the manuscript.

## Declaration of interests

All authors have no conflict of interest.

## Supplemental information

Document S1. Figures S1–S3 and Tables S1–S3

Document S2. Article plus supplemental information
