# Supplementary material for: CRISPR-Cas9-mediated homology-directed repair rescues the induced bone marrow failure in Fancc−/− mice
Source: Mol Ther Nucleic Acids. 2026 Mar 23;37(2):102912. doi: 10.1016/j.omtn.2026.102912 (PMC13087461; doi:10.1016/j.omtn.2026.102912)
Supplement: Document S1. Figures S1–S3 and Tables S1–S3 [file mmc1.pdf]

OMTN, Volume *37*

Supplemental information

CRISPR-Cas9-mediated homology-directed repair rescues the induced bone marrow failure in *Fancc*$^{-/-}$ mice

Hemavathy Harikrishnan, Mahesh Lamsal, Ka-Kui Chan, Junping Zhang, Jiahe Tian, Kwadwo Fosu, Hong Phuong Nguyen, D. Wade Clapp, Elizabeth A. Sierra Potchanant, Reuben Kapur, Weidong Xiao, and Ngoc Tung Tran

Figure S1

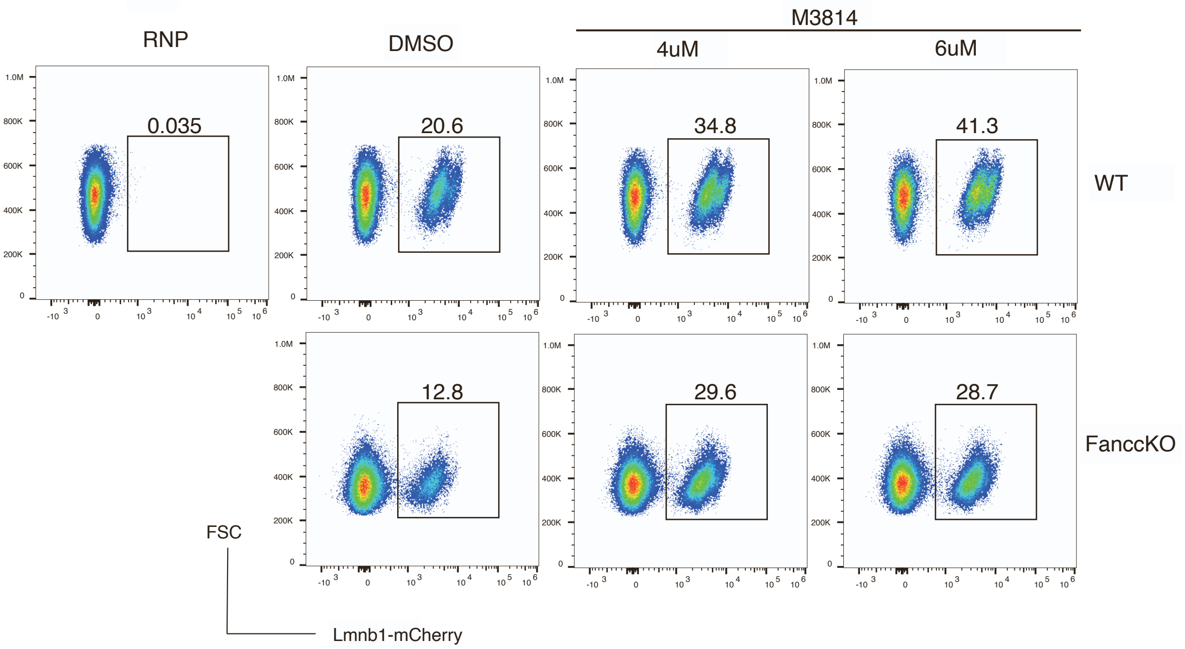

Figure S1. Flow cytometry showing the efficiency of HDR in wildtype (WT) and Fancc-/- (FanccKO) cells treated with CRISPR/Cas9. Cells were treated with different concentration of M3814 (DNA-PKc inhibitor). Percentage of mCherry cells reflects the HDR efficiency in the Lmnb1 locus.

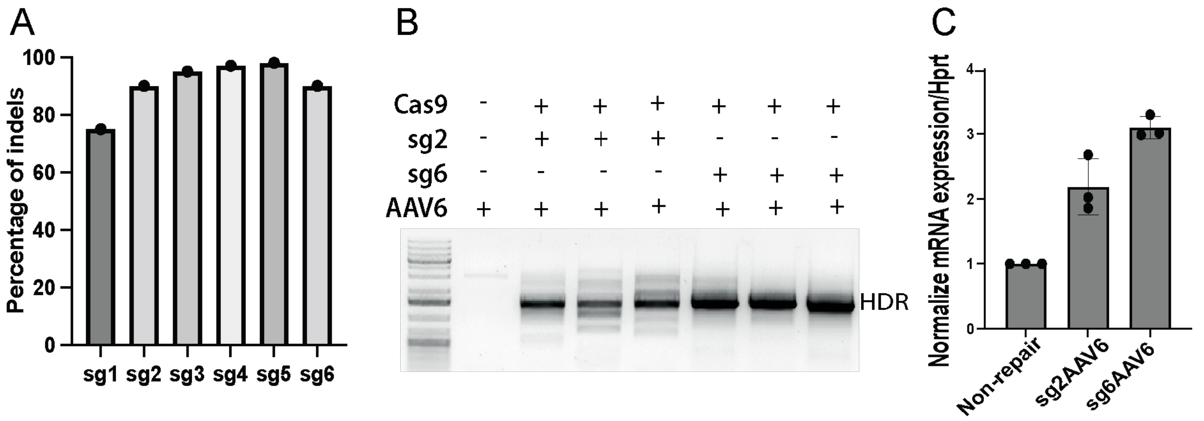

Figure S2. (A) Cutting efficacy of different sgRNA targeting the exon 2 of Fancc locus. (B) PCR to detect the knockin event (HDR) in cells treated with gene editing system using 5HA forward primer (outside of homology arm) and reverse primer annealing to Fancc cDNA. (C) Real-time PCR showing the expression of Fancc mRNA in corrected stem cells using two different sgRNAs.

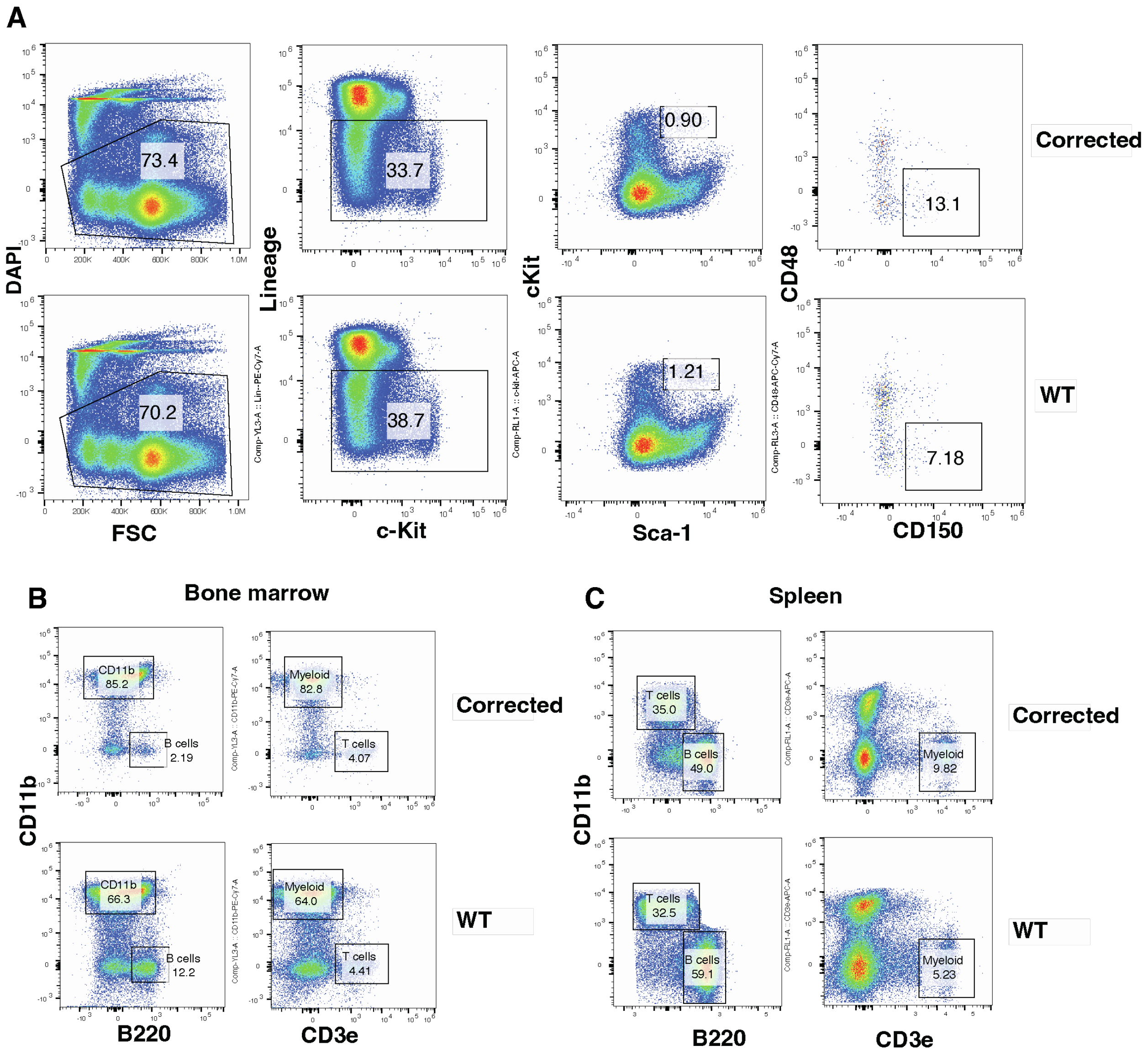

Figure S3. Flow cytometry analysis of 2nd transplanted mice. (A) gating strategy for LSK and HSC/MPP1 compartment. Gating strategy for myeloid (CD11b), B cells (B220) and T cells (CD3e) in the bone marrow (B) and spleen (C).

Supplemental Tables

Table S1. Potential off-target sites predicted by CrispRGold.

sgRNA_6				
GTATGAAATCTTGAAGGAGATGG	sequence of sgRNA			On Target
**Top off-target sites**	**Position**	**Annotation (if relevant)**	**Risk**	
C..............C...A..	chr8:108192534:108192555	Intergenic	high	OF1
a..-...............a..	chr3:88477414:88477434	Intergenic	high	OF2
a..-...............a..	chr9:53505828:53505848	ATM.Intron;ATM.RNA.Intron	high	OF3
...-.t.............a..	chr18:22746269:22746289	NOL4.Intron	high	OF4
........T....G.....G..	chr6:140426687:140426708	PLEKHA5.Intron;PLEKHA5.RNA.Intron	high	OF5
...........c...a...a..	chr5:57077893:57077914	Intergenic	high	OF6
a..a...........a...a..	chr6:4672781:4672802	Intergenic	high	OF7
c.AT...............a..	chr3:88477413:88477434	Intergenic	high	OF8
..CC...........A...A..	chr16:3257952:3257973	Intergenic	high	OF9
.GG..G................	chr14:109505973:109505994	GM30216.Intron	high	OF10
...-........T......g..	chr10:58386743:58386763	LIMS1.Intron	high	OF11
...........t......ga..	chr5:93330133:93330154	Intergenic	high	OF12
.G............A..A....	chr7:96606604:96606625	TENM4.Intron	high	OF13
..g.g..........a...g..	chr6:22830601:22830622	Intergenic	low	OF14

Table S2. Oligos used in the study

Name	Sequence
Sg2	GACAGTGCTGCTCTCAGAGA
Sg6	GTATGAAATCTTGAAGGAGA
mFancc_T7For	caccgttagtgagttcctgtggaag
mFancc_T7Rev	Tagctaacatgctgaagaccctggg
mFancc_5HA_ext_F1	ctgcccttctctacaggggcactat
mFancc_3HA_ext_R1	caccattgaagagaggcccctccat
mFanccCDNA_for_4.1	ggcccacacggatgctgtaatccat
mFancc_RT_5F	GCTGAGGGAGGAAGCTGAGC
mFancc_RT_5R	AGGCGTCTGACCAGCTGTCC
mHprt_RT_F	CGTCGTGATTAGCGATGATG
mHprt_RT_R	ACAGAGGGCCACAATGTGAT

Table S3. Antibodies used in the study

Antibodies	Company	Catalog number
CD3e-APC	BioLegend	152306
PE anti-mouse Gr-1 (Clone RB6-8C5)	BioLegend	108407
PE/Cy7 anti-mouse CD11b (Clone M1/70)	BioLegend	101215
BV785 anti-mouse B220 (Clone RA3-6B2)	BioLegend	103245
Biotin anti-mouse/human CD45R/B220 Antibody (Clone RA3-6B2)	BioLegend	103204
Biotin anti-mouse/human CD11b Antibody (Clone M1/70)	BioLegend	101204
Biotin anti-mouse Ly-6G/Ly-6C (Gr-1)(Clone RB6-8C5)	BioLegend	108404
Biotin anti-mouse CD3ε Antibody (Clone 145-2C11)	BioLegend	100304
Biotin anti-mouse TER-119 (Clone TER-119)	BioLegend	116204
PE/Cyanine7 Streptavidin	BioLegend	405206
BV785 anti-mouse Ly6A/E (Sca-1) (Clone D7)	BioLegend	108139
APC anti-mouse CD117 (c-kit) (Clone 2B8)	BioLegend	105812
BV605 anti-mouse CD150 (Clone TC15-12F12.2)	BioLegend	115927
APC/Cy7 anti-mouse CD48 (Clone HM48-1)	BioLegend	103431
